# Rethinking the history of microbiology: new actors, geographies, places of knowledge, and ecologies

**DOI:** 10.1007/s40656-025-00678-2

**Published:** 2025-06-23

**Authors:** Matheus Alves Duarte da Silva, Mathilde Gallay-Keller

**Affiliations:** 1 University of St Andrews, St Andrews, Scotland; 2 Musée des Confluences, Lyon, France

**Keywords:** Historiography, Bacteriology, Louis Pasteur, Bruno Latour

## Abstract

In this introduction, we first paint a panorama of the historiography of microbiology from the end of the nineteenth century until today, spanning from Pasteurian hagiographies, institutional histories, STS-informed analyses to critical research on the emergence of microbiology in an age of global empires. We then suggest three possibilities for historians and anthropologists to rethink the past and present of microbiology: (1) by centralizing the focus of their analyses on geographies and actors outside of the realm of the Pasteur Institute and of the Pasteurians; (2) by studying places of knowledge beyond the laboratory and their interactions with the laboratory; and (3) by researching the past and present of complex ecologies that go beyond sole interactions between humans and pathogenic microbes. These three ways of recentralizing the history of microbiology are not unprecedented nor were they firstly enacted in this collection. On the contrary, this collection builds on a growing stream of innovative research and widens current avenues of research, helping thus to rethink the history of microbiology in a more global and inclusive way.

Microbes are great again—at least for the humanities. From ethnographies of microbial ecologies in a post-Pasteurian world^1^ (Paxson & Helmreich, 2014), epistemological investigations into humanity’s capacity to change bacteria’s inner structure (Landecker, 2016), historical examinations on the role of microbes in maintaining or compromising imperial infrastructures (Velmet & Kirchhelle, 2024) to studies placing discoveries on microbes in different scientific traditions (Grote, 2025; Strasser, 2019), social and cultural studies on microbes are pullulating. This mushrooming interest stems from the perception that microbes occupy an ambivalent place in modern, industrialized, and capitalistic societies. Whilst their centrality in the making of antibiotics, other drugs, and agricultural products, as well as more mundane objects like alcoholic beverages and fermented food, has skyrocketed in the past decades, pathogenic microbes bore the threat of dystopian cataclysmic scenarios in which humanity collapses, as the Covid-19 pandemic gave an *avant-gout*. Microbes have thus become *good to think with* because of their ambiguity, at the edge of nature and culture, visible and invisible, friends and foes.

Aiming to take stock of this contemporaneous research in the humanities and to encourage new ways of writing the history of microbiology and of the links between humans and microbes, we organized the online seminar “Microbes and Microbiology: Towards  New Stories?” in spring 2021. Our goal was then to map the questions, objects, methods, and approaches currently shaping our understanding of the past and present of microbiology and of the technical and social changes it has spawned until the present day. The webinar reunited an international team of historians, anthropologists, sociologists, and philosophers.^2^

Extending the fruitful discussions raised during the event, this collection reflects on the history of microbiology from the end of the nineteenth century until the early twenty-first century and proposes approaching it under three often-ignored perspectives. These perspectives, explained in further detail in the last section, could be summarized as follows. First, we suggest rethinking the history of microbiology by moving the focus towards actors and spaces outside, or at the edge of, the realm of Louis Pasteur and the Pasteurians. Such a suggestion does not deny the role of Pasteur, the Pasteur Institute, and its network of overseas laboratories to the construction and consolidation of microbiology. After all, in this introduction we will use the term “microbiology” as crafted by Pasteurians (see below). This suggestion only intends to cast light on understudied actors and geographies. Second, and partially linked to the first point, we suggest rethinking the history of microbiology by moving the focus from the laboratory to other places of knowledge^3^ on microbiology—this may refer, among other places, to hospitals, veterinary clinics, museums, cheese factories, and garage laboratories. Finally, we suggest rethinking the history of microbiology by moving the focus from past and present research on pathogenic microbes to past and present research on complex ecologies in which microbes, whether pathogenic or not, are one element among others.^4^ Therefore, rather than decentralizing the history of microbiology, this collection aims to centralize it in new actors, geographies, places of knowledge, and ecologies. At a methodological level, this collection is a call to interdisciplinarity, by demonstrating the benefits for historians to seize insights from the ethnography and for anthropologists to go to the archives and mobilize the tools of the history of science and medicine.

The term “microbiology”^5^ [*microbiologie*] was used in France for one of the first times in 1883 in an encyclopaedic entry written by Émile Duclaux. Alongside his friend and master Louis Pasteur, Duclaux had been developing a new scientific discipline called “biological chemistry” [*chimie biologique*].^6^ In Duclaux’s words (1883, p. 1), “biological chemistry” comprised the study of “all phenomena, both chemical and physiological, in which organised cells, always deprived of chlorophyll, called ferments, intervene as active agents”. These beings only visible under the microscope had been discovered to be central for several human activities, such as the fermentation of beer and cheese, but also for spoiling food and beverages. Duclaux admitted that the term “biological chemistry” was “poorly chosen; microbiology [*microbiologie*] would be much better”. But he justified keeping it so to facilitate encyclopaedic classifications (Duclaux, 1883, p. 7). Yet a few years later the word microbiology replaced biological chemistry, appearing in the title of Pasteur Institute’s official journal, edited by Duclaux, the “*Annales de l’Institut Pasteur: journal de microbiologie”* first printed in 1887, and in Duclaux’s *Traité de Microbiologie,* published in 1898. In this handbook, Duclaux placed the main interest of microbiology not only in the study of ferments, but rather in that of *all* microbes, which he defined as “beings devoid of chlorophyll and any other protoplasmic pigment […] single-celled and visible only under the microscope” (Duclaux, 1898, p. 56). According to Duclaux, microbes were not only useful beings to the industry, but could also be pathogenic to plants, animals, and humans. Nonetheless, some of them could be manipulated in laboratories, their virulence progressively attenuated, and vaccines and treatments created against them. In Duclaux’s view, microbiologists’ main work would be to identify the specific microbes responsible for infectious diseases, and strive to find a cure or a way to prevent against the maladies they provoked (Duclaux, 1898, p. 608).

Without any normalizing intention and without entering into the debate whether microbes exist without microbiology (Demeulenaere & Lallier, 2024), we understand microbiology here close to Duclaux’s definition, as the science whose object of study is the microbe, pathogenic or not, broadly defined. Also, we understand microbiology as a science usually split in two fronts: study and manipulation of microbes for producing food and beverages, on the one hand, and study and manipulation of microbes for producing vaccines, sera, and other drugs to fight diseases caused by microbes in plants, animals, and humans. The historiography of microbiology has usually sidelined the first aspect, concentrating on the medical side. Nevertheless, this gap has been partly addressed in the last years thanks to innovative studies on fermentation (Lee, 2015; Paxson & Helmreich, 2014). In this special issue we strove to bridge together the medical and alimentary aspects of microbiological research, but we did not achieve a perfect balance.

In what follows we first paint an impressionist panorama on the historiography of microbiology in the last century. Departing from an impossible view-from-nowhere, we chose instead to foreground our analysis on publications in and about our fieldworks—Brazil and France—addressing our main academic interests—global histories of microbiology and histories and ethnographies of microbial collection in France and Europe—and focusing on the years between 1880 and 1920, which correspond to the global emergence of microbiology. Then, we discuss our claim for recentring the history of microbiology and demonstrate how the five papers empirically provide new avenues of investigation.

## From Geniuses to Empires

First accounts on the history of microbiology were usually written by friends and relatives of important microbiologists or by the microbiologists themselves. In France, these stories recounted the life and genius of Pasteur, and later, of Pasteurians, such as Albert Calmette and Alexandre Yersin.^7^ Worldwide, similar biographies/hagiographies sprung up regarding Pasteur’s counterparts, like Oswaldo Cruz, commonly dubbed as the “Brazilian Pasteur” (Britto, 1995). As noticed by Geison (2014, p. 278), “these heroic hagiographies were used to transmit widely accepted moral verities and in which science was seen as straightforwardly useful and ‘positive’ knowledge”.

Professional humanities scholars became more interested in the history and social aspects of microbiology in the post-war period. Nancy Stepan’s study on the Laboratory of Manguinhos in Rio de Janeiro, Brazil, is a sound example of these first waves of humanistic studies on microbiology (Stepan, 1976). Stepan’s work was directly influenced by George Basalla’s three-step model on the transfer of modern science from Europe to the Rest but also from examinations on the emergence of innovative research in countries located at the periphery of capitalism like Brazil. According to Stepan, the Laboratory of Manguinhos, created in 1900, became a model of success in Brazil thanks to the capacity of its director Oswaldo Cruz in adapting the managerial model of the Pasteur Institute in Paris—cutting-edge research, teaching, and industry—to the Brazilian reality. Therefore, Stepan argued that Manguinhos’ discoveries on tropical diseases, mainly the identification of Chagas Disease in 1909, marks the beginning of a Brazilian national tradition of scientific research.

In 1984, Bruno Latour depicted an extremely dynamic history of microbiology, going beyond institutional narratives such as Stepan’s. In a now-classic book laying the foundations of the STS, Latour described with a Machiavellian prose the emergence of microbiology in France as resulting from the ability of Pasteur and his disciples to forge new alliances between humans and microbes and translate the interests of both groups. For Latour, this translational work took place inside Pasteur’s laboratory, and became embodied in the form of vaccines, sera, and other objects. From Pasteur’s laboratory, these objects pasteurized—e.g. they reconstructed—first France and then the French colonies in Africa and Asia, although Latour never provided a substantial analysis of how microbiology took root outside of France (Latour, 1984). In short, far from desacralizing the scientific genius of Pasteur, Latour transformed Pasteur into a tactical and political genius. Latour has also contributed to the centrality of the laboratory in the social studies of microbiology, still evidenced by the terminology of “laboratory ethnography”.^8^

Latour’s approach to the history of microbiology in France was adapted to other national contexts. For example, the sociologist of science Henrique Cukierman (2007) showed how Oswaldo Cruz and his inner circle of disciples at Manguinhos managed to Pasteurize Brazil in the first half of the twentieth century, in which the interests of Brazilian elites and those of microbes, but also of mosquitos, were translated at Manguinhos in the form of artefacts and sanitary practices like vector destruction. Thus, if the world became Pasteurized at the turn of the twentieth century, the history of microbiology became *Latourized* one hundred years later.

Despite its global range, Latour’s thesis was never unanimously received in France. The historian Ilana Löwy (2010, 2015) argued that the idea of the Pasteurian Revolution should be relativised and perhaps even abandoned: in early twentieth-century France, microbiology appeared rather as a technology of hope than a game-changer, and its promises of taming microbes for putting them into the service of society were far from being attained. Löwy also contributed to a better understanding on how microbiology circulated outside of France, mainly in Brazil. In a work that is better appreciated if read in tandem with Benchimol (1999), Löwy demonstrated that microbiology and the microbiological revolution—if there was one—emerged in Brazil not thanks to the sole genius of Cruz and his laboratory, but amidst a social, political and scientific process of fighting the yellow fever. In this process, the search for yellow fever’s microbe and anti-mosquito campaigns were strictly connected to modernization and centralization projects of Brazilian elites and to the circulation of knowledge between Brazilian doctors, Pasteur Institute and North-American experts (Löwy, 2001).

This last point—the transnational transfer of the Pasteurian sciences—has been a perennial subject of interest for historians of microbiology. They have particularly focused on the foundation of overseas Pasteur Institutes or on scientific missions carried out by scholars attached to the Pasteur Institute in Paris, arguing that these laboratories and actors helped to export microbiological theories and practices, as well as European moral paradigms of hygiene and health (Chakrabarti, 2012; Löwy, 1990; Moulin, 1996, 2016).

Lastly, this transnational history of microbiology has gained new dimensions thanks to postcolonial, decolonial, and circulatory approaches. The recent work of Chien-Ling Liu and Aro Velmet exemplifies this new trend of scholarship, as well as their tensions. Liu (2017) paid particular attention to the manufacturing and distribution of smallpox vaccine in the Pasteur Institute of Chengdu in China. Liu argued that French doctors adapted Pasteurian medicine to Chinese medical traditions and environment, exemplified in the selection of local cattle to produce the lymph and vaccinating using traditional practices of variolisation and acupuncture. Velmet, on the other hand, discussed the capacity of Pasteurians like Albert Calmette to use French colonies as a laboratory for imposing and developing Pasteurian medicine, arguing that the new science of microbes became instrumental to rule the French empire. This ever-growing scientific, economic, and political enterprise, illustrated by the expanding network of overseas Pasteur Institutes, Velmet (2020) dubbed the Pasteur’s Empire, although Louis Pasteur himself contributed little to forging it.^9^

In short, from analyses centred on the genius of Pasteur and his counterparts like Cruz, and then lately studies on the ability of microbiologists to change science and society by forging alliances between humans and microbes within the laboratory, historians and anthropologists of microbiology have now moved towards a critical stance on the legacy of Pasteur and the Pasteurian Revolution or towards more nuanced accounts on the globalization of microbiology. From then on, what are the new research avenues for more complex and inclusive histories of microbiology?

## Recentring the History of Microbiology

The recent historiography on the global career of microbiology has usually understood the world as split between two main poles: an imperial core and a wide periphery. In this narrative scheme, microbiological knowledge emerged and was crafted usually in France, Germany, and, on a smaller scale, in Britain, and then exported, contested, adapted, or circulated to a large Rest spanning from Senegal to India, from Brazil to China. If nobody can deny the decisive role played by Pasteur, France, and Europe in the history of microbiology, the historiography of the global career of microbiology often misses the density of the Rest’s landscape and, most importantly, that microbiology rose in a world of complex geopolitics. For example, non-European empires not only existed in the late nineteenth century (Japan, China, but lest we forget Brazil), but some deployed microbiology ideas and practices to expand or consolidate their territories (Gomes 2013; Nosaka & Silva 2023). Conversely, the educated elites of independent South American republics like Argentina, Peru, and Brazil (post-1889), learned with microbiology how to tame tropical diseases and capitalized political gains upon these scientific victories (Cueto, 1997; Kropf, 2009, and Zabala & Rojas, 10.1007/s40656-024-00648-0). Furthermore, as one of us has showed, microbiological knowledge not only circulated between Europe and the Rest, but within the Rest (Silva 2018; Nosaka & Silva 2023). Finally, historians have showed that microbiological knowledge crafted in the Global South, and at times by non-European actors, was adapted to Europe (Gradmann, 2010 and Silva & Goodman, 10.1007/s40656-024-00633-7) or that microbiological knowledge developed in the Global South challenged dominant visions of microbiology governance (Haraoui, Rizk & Landecker, 10.1007/s40656-024-00624-8).

Therefore, the first recentralization that we would like to suggest is a geographical one, e.g., moving the focus away from dominant European institutions, above all the Pasteur Institute. All articles assembled here focus elsewhere from the Pasteur Institute in Paris, and in most cases, from overseas Pasteur Institutes. This choice allows the discovery of microbiological knowledge emerging in a varied range of geographic locations often ignored in English-speaking histories of microbiology, including Tokyo, Algerian and Argentinian rural areas, Beirut, and the French Jura region. However, a mere geographic recentralization would not represent a great novelty. After all, historians have written about microbiology being imposed or adapted outside of Europe or emerging in non-European countries like Brazil. Henceforth, a central aspect for this special issue is to suggest possibilities to rethink patterns of scientific exchanges on microbiology in which knowledge crafted in the Rest is adapted in Europe (Silva & Goodman), that places in the Rest take paths away from the research carried out in the North (Nosaka; Zabala & Rojas; Haraoui, Rizk & Landecker), or that scientific disagreements emerge within the North (Demeulenaere).

Alongside a geographic recentring, this special issue aims to rethink the history of microbiology by focusing on places of knowledge different from, and on their interactions with, the laboratory. As showed above, the laboratory has occupied a prominent place in historical and ethnographic analyses on microbiology either in Europe or in the Rest. The laboratory has been understood as the place par excellence to craft microbiological knowledge or to knit new alliances between humans and microbes. This almost exclusive choice often eclipses the role of other places of knowledge on microbiology or makes them appear to be in the background. But although not dominant, an extra-laboratorial or multi-sited approach exists in the history and the anthropology of microbiology, with researchers paying attention to collections in the emergence and development of microbiology (Brives 2022; Gallay-Keller 2021; Strasser, 2019), the influence of industrial skills in laboratory activities (Gaudillière & Löwy, 1998), the connections between farms, stables, and laboratories (Simon, 2008), and the role of ships in the development of contagium theories and disinfection practices (Engelmann & Lynteris, 2019). The articles in this special issue build on these previous studies and stress how new knowledge emerged on alternatives places of knowledge, such as hospitals in Lebanon and Japan (Haraoui, Rizk & Landecker; Nosaka), research stations in rural areas of Argentina and Algeria (Zabala & Rojas; Silva & Goodman), and cheesemaking workshops in the French countryside (Demeulenaere).

Nonetheless, as a hospital in Beirut during the Lebanese civil war is different from a hospital in Meiji Era Tokyo, the articles do not simply recentralize their focus on alternative places of knowledge, but reflect on the social, political, and ecological specificity of each of them. Developing this last point, as for the third recentralization we would like to suggest rethinking the history of microbiology from an almost exclusive focus on microbes interacting with humans to more holistic and ecological analyses. The study of microbes, namely pathogenic ones, has entrusted microbiology with a significant academic and social identity since the end of the nineteenth century. And one does not need to be Latourian to acknowledge that microbes are relevant in any social and cultural study of microbiology. Therefore, to scrub pathogenic microbes out from the history of microbiology would be useless, not to say nonsensical. Our claim here is more in line with recent works of historians and anthropologists, who have studied complex ecologies in which microbes—pathogenic or not—are one element among others (Gallay-Keller, 2021; Keck, 2020; Lynteris, 2022; Paxson & Helmreich, 2014). This collection discusses the elements involved in the production of terroir cheese in France (Demeulenaere); in attenuated forms of malaria and plague in Algeria, France, and Argentina (Silva & Goodman; Zabala & Rojas); in antimicrobial resistance in hospitals in Beirut (Haraoui, Rizk & Landecker), and in anti-cholera treatment failures in Tokyo (Nosaka).

To sum up, this collection explores three ways of rethinking the history of microbiology, which can be translated into three recentralizations: a geographic recentralization, moving beyond Europe or key European institutions; an institutional recentralization, going beyond the laboratory as the main place for producing microbiological knowledge; and a thematic recentralization, going beyond sole interactions between humans and pathogenic microbes, and taking these interactions as part of complex ecologies in which other elements are involved. The articles assembled explore in detail one or many of these three ways of rethinking the history and the anthropology of microbiology.

Opening the special issue, Shiori Nosaka’s *Inventing with Bacteriology* reinterprets an episode usually seen as a failure in the career of Japanese bacteriologist Kitasato Shibasaburo: the experimental administration of anti-cholera serum during the 1895 outbreak. This series of treatments was a critical stance against German bacteriologist Robert Pfeiffer’s claim that serotherapy could be more dangerous to cholera patients than regular treatments. In the Tokyo Hiro’o Quarantine Hospital, Kitasato underwent an innovative “therapeutic trial” and considered that the results probed the anti-cholera serum’s efficiency. Yet Japanese and German scientists contested it. In the end, Kitasato lost the controversy, and the anti-cholera serum disappeared from Japanese and German debates. Departing from analyses of successes or failures, Nosaka’s article contributes to understanding the role of non-European actors and extra-laboratory places of knowledge in the early global history of microbiology.

In *Of Rats and Children*, Matheus Duarte da Silva and Jordan Goodman investigate the early history of the concept of disease reservoir. The article starts with the 1922 claim by Louis Tanon that the rats of Paris were a plague reservoir, which reshaped the fight against plague in the French capital. Tanon’s conclusion found its roots in studies by British experts on colonized children as sources of malaria in Sierra Leone, and in the research by the Sergent brothers on children as reservoirs of malaria in rural Algeria, when the concept of reservoir gained one of its first medical dimensions. Tanon was also indebted to studies carried out in India on the capacity of rats harbouring the plague bacillus without being killed by it. Going against the historiographical grain, Silva and Goodman describe thus how research on rats and malarial reservoirs invented in the Rest was applied to fight plague in France.

*From the ports to the hinterlands* brings the collection to Argentina. Juan Zabala and Nicolas Rojas bridge together two moments of plague in that setting often described in isolation. Firstly, the period between 1899 and the 1910s, when a first generation of Argentinian microbiologists demonstrated that plague was mild when treated with massive doses of serum in studies conducted in laboratories and hospitals in Buenos Aires. Secondly, the 1920s and 1940s, marked by research on the endemicity of plague in rural areas of the provinces of Jujuy and Salta. These investigations demonstrated that plague in the Argentinian countryside had a much more complex ecology, a condition that became known as rural plague. These two periods reveal that the “internalization” of plague in Argentina was followed by an expansion of both the Argentinian state and microbiologist’s range of action towards the hinterland. Moreover, the article demonstrates original microbiological ideas and practices emerging in urban and rural places of knowledge of a non-European country.

In *States of Resistance,* Louis-Patrick Haraoui, Anthony Rizk, and Hannah Landecker investigates how global, meso-, and micro levels wove together in processes of Antimicrobial Resistance (AMR) in Beirut’s American University Hospital. The authors depart from USA- and UK-centred One Health and AMR approaches, which usually frame situations like those in the Beirut hospital as of a lack of hygiene or failure to abide to international protocols. Instead, Haraoui, Rizk, and Landecker take the social, ecological, and microbial local contexts historically seriously. They demonstrate that the Lebanese civil war (1975–1990) shaped the complex microbial environment of the American University Hospital, leading to an upsurge in the *Acinetobacter baumannii* as a nosocomial threat. This case study is thus another example of the biology of history as theorized by Landecker (2016, p. 21), in which “human historical events and processes have materialized as biological events and processes and ecologies”.

Closing the special issue, Élise Demeulenaere’s *In Search of the Microbial Path to Terroir* examines the history of French cheese microbiology in the last decades of the twentieth century. Focusing on cheesemakers, entrepreneurs, and scientists gravitating around laboratories and research groups located mainly in the French countryside and linked to Protected Designation of Origin (PDO) cheeses, Demeulenaere showcases how these actors chose to value terroir, understood as the complex relation between taste and place. This valorisation resulted in a comprehension of cheese as more than just the effect of fermenting starter cultures, and enhanced studies on the native “microflora reservoirs”, which included disparate elements such as hay, udders, meadows, and the “atmosphere of the milking parlor”, and on microbial fluxes from farm to fork. Demeulenaere’s article reveals thus a burgeoning world of microbiological knowledge production in rural areas, leading to the pioneering emergence in France of a new field: microbial ecology applied to the cheese industry.

To conclude, we insist that these three ways of recentralizing the history of microbiology are not unprecedented nor were they firstly enacted in this collection. On the contrary, this collection builds on a growing stream of innovative research in the humanities focusing on microbes and microbiology. Therefore, these ways of recentralizing should be understood as widening current avenues of research, so to help to rethink the history of microbiology in a more global and inclusive way.

## Data Availability

Not applicable.

## Footnotes

1 For a criticism on the Post-Pasteurian and microbial turns, see the special issue directed by (Brives & Zimmer, 2021).

2 For the event’s argument and line-up, see https://wwrat.wp.st-andrews.ac.uk/events-2/.

3 On places of knowledge, see (Jacob, 2017).

4 Regarding this third point, our approach shares a similar endeavour with a recent special issue published in this journal (Honigsbaum & Méthot, 2020). However, while this other collection reflects on medical aspects of ecological thinking in biomedicine, ours also pays attention to the ecology of non-pathogenic microbes.

5 We have chosen the word microbiology in this introduction, but some authors in this collection have preferred the term bacteriology. This decision stems from national and institutional traditions on naming the science(s) of microbes.

6 On Duclaux, see (Löwy 2010, pp. 205–208).

7 For examples on Pasteurian biographies and, some of them, hagiographies, see (Noël Bernard, 1961; Mollaret & Brossollet, 1985).

8 For critical examinations on Latour’s seminal work, see (Demeulenaere & Lallier, 2024; Schaffer, 1991).

9 Rather, it was shadowy Pasteurians who crafted this “empire”. Also, these sometimes-overlooked actors helped to centralize microbes from all over the world in the microbial collections of the Pasteur Institute in Paris (Gallay-Keller, 2021).
